# Trastuzumab therapy duration in HER2-positive de novo metastatic breast cancer: 1999–2018

**DOI:** 10.1007/s10549-022-06678-1

**Published:** 2022-07-22

**Authors:** Henry G. Kaplan, Judith A. Malmgren, Boya Guo, Mary K. Atwood

**Affiliations:** 1 Swedish Cancer Institute, 1221 East Madison, Seattle, WA USA; 2 HealthStat Consulting, Inc., Seattle, WA USA; 3 School of Public Health, University of Washington, Seattle, WA USA

**Keywords:** Metastatic breast cancer, Survival, Complete response, HER2, Trastuzumab, Treatment duration

## Abstract

**Purpose:**

The optimal duration of first-line trastuzumab (T) treatment for de novo stage IV HER2-positive metastatic breast cancer (MBC) patients after complete response (CR) is not known.

**Methods:**

A retrospective cohort study of de novo stage IV HER2-positive MBC patients who had trastuzumab included in their initial treatment (*n* = 69), 1999–2018, was conducted with follow-up for CR, progressive disease (PD), vital status, and disease-specific survival (DSS). Statistics included Kaplan–Meier plots and Cox proportional hazards models.

**Results:**

Mean trastuzumab treatment time was 4.1 years (range 0.1–15). 54% of patients experienced CR at average time 9 months on treatment (*n* = 37). Eight CR patients discontinued T treatment after 18 months average post-CR time (range 0–86) and twenty-nine stayed on T treatment post CR [average 65 months (range 10–170)]. Average follow-up was 6 years, range 1–15 years. 5-year DSS was 92% for CR on T patients (*N* = 29); 88% CR off T (*n* = 8); 73% No CR on T (*n* = 14); and 29% No CR off T (*n* = 18) (*p* < 0.001). In forward Cox proportional hazards modeling, CR = yes [HzR = 0.31, (95% CI 0.14, 0.73), *p* = 0.007], continuous T treatment > 2 years [HzR = 0.24, (95% CI 0.10, 0.62), *p* = 0.003], and age < 65 [HzR = 0.29, (95% CI 0.11, 0.81), *p* = 0.018] were significantly associated with better DSS.

**Conclusion:**

Maximum trastuzumab treatment time to CR was 27 months with 2 or more years trastuzumab treatment independently associated with better survival. Survival comparisons and hazard modeling both indicate as good or better survival associated with continuous trastuzumab treatment regardless of CR status.

Word count (*n* = 250).

## Introduction

Five percent of newly diagnosed breast cancers are stage IV metastatic breast cancer (MBC) with 26% of them human epidermal growth factor receptor 2 (HER2) positive [1]. Most HER2-positive MBC patients are currently treated with trastuzumab combined with chemotherapy such as a taxane and often pertuzumab as first-line therapy [2]. First-line trastuzumab-treated HER2-positive MBC patients have significantly improved survival [3]. Achievement of complete response (CR) status has afforded a long-term survival outcome unexpected with a de novo metastatic breast cancer diagnosis [4].

The NCCN recommendation for duration of adjuvant trastuzumab treatment of non-metastatic invasive HER2-positive breast cancer is 12 months [5]. The American Society of Clinical Oncology (ASCO) guideline for HER2-positive advanced/metastatic breast cancer trastuzumab treatment is to continue HER2-targeted therapy without change unless there is progression or unacceptable toxicities [6]. From a hospital registry study and case studies, some have suggested it is possible to discontinue HER2-positive MBC trastuzumab therapy after CR is achieved without deleterious effect, though optimal length of treatment in the metastatic setting remains unclear [7–10].

The optimal duration of trastuzumab treatment in HER2-positive de novo MBC is not known. The objective of our real-world retrospective cohort study was to assess the relationship between trastuzumab treatment duration and patient outcomes among de novo MBC HER2-positive patients at the Swedish Cancer Institute. Factors evaluated were trastuzumab continuation/discontinuation, trastuzumab treatment duration, CR, relative breast cancer-specific survival (DSS) by treatment/CR category, and proportional hazard of mortality associated with CR and trastuzumab duration.

## Methods

For our retrospective cohort study, we used patient data from our institutions’ breast cancer research registry database maintained continuously since 1995 with data collected on all patients. Study eligibility criteria were (1) diagnosis of HER2-positive de novo MBC, (2) diagnosis year 1999 to 2018, (3) treatment naïve first primary breast cancer diagnosis, and (4) receipt of trastuzumab as part of initial first-line therapy. Of 12,786 patients in the registry from 1999–2018, 337 patients were diagnosed with de novo MBC and 82 of these cases were HER2-positive. Patient diagnosis year for inclusion was 1999 when HER2 testing and trastuzumab treatment began and truncated at 2018 to allow for minimum potential follow-up of 2 years through 2020. Ten of the 82 HER2-positive MBC patients did not receive trastuzumab treatment and three patients were lost to follow-up. Sixty-nine patients met eligibility criteria for inclusion.

Incident BC cases were entered at time of diagnosis into the HIPAA compliant and IRB-approved registry [IRB Study ID SWD39425-03]. This study was issued exemption status and approval by the Swedish Cancer Institute IRB program as the data are de-identified for analytic and study purposes. Patient vital and disease status including date and cause of death are collected by annual updates by certified cancer registrars complete through 2020 for this cohort. If cause of death was not determined from the chart, death certificates were obtained. Date of death from breast cancer was the endpoint for disease-specific survival (DSS).

Starting and ending treatment dates are tracked in the registry database for pre-operative chemotherapy as well as other chemotherapy, anti-HER2-directed antibody therapy, hormonal therapy, and radiation therapy. Continuous systematic follow-up and review of patient electronic medical records in the institutional registry database are conducted at regular intervals. Follow-up status is obtained from annual updates by (1) electronic chart review, (2) IRB-approved physician directed follow-up letter, (3) the institution’s cancer registry, and (4) SEER Seattle-Puget Sound Registry [11].

Oligometastatic disease was defined as 1–2 distant metastatic sites. Hormone receptor (HR) positivity was estrogen and/or progesterone receptor-positive (HR-positive) and HR-negative if negative for both. If only estrogen or progesterone were positive at 10% or less, HR status was marked as negative [12]. Self-reported race was coded white/non-white. HER2 positivity was determined by immunohistochemistry with weak positive (2 +) confirmed by FISH test (fluorescence in situ hybridization) [13].

**Fig. 1:**
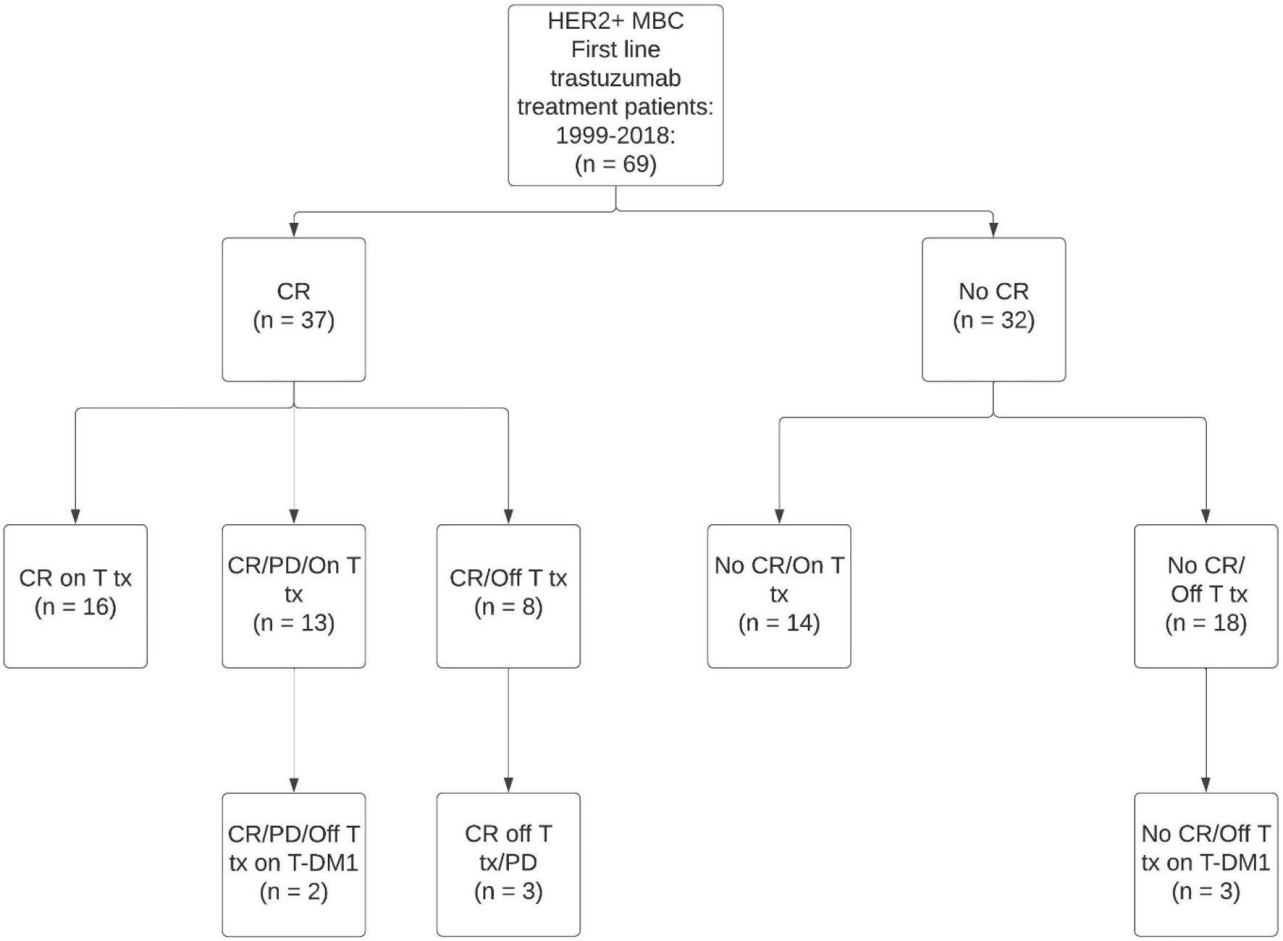
Consort diagram

If a patient had clinical and radiologically confirmed complete response with no evidence of disease (NED), CR was recorded on that date. Progressive disease (PD) was recorded by the same method. Complete response (CR) was assigned if the absence of clinical disease was observed in an imaging study or studies and the treating physician confirmed the absence of disease by clinical examination. Imaging was prompted by clinical presentation (signs/symptoms) at a regular scheduled check-up or by a visit scheduled by the patient for symptomatic or observable physical complaint. Progressive disease (PD) was assigned if disease was observed in imaging studies and confirmed by the treating physician. (Fig. 1: consort diagram).

Time intervals for each patient’s individual experience of time on and off trastuzumab treatment, and time to outcomes were calculated including time to CR, time post CR [Duration of Response (DOR)], time from CR to PD, time to subsequent CR post PD, and time post PD. Combined treatment and outcome (continuous vs. discontinuous treatment and complete remission yes/no) were used to create four analytic categories: (1) CR on T treatment/T treatment discontinued after CR (CR off T treatment); (2) CR on continuous T treatment (CR on T treatment); (3) No CR on continuous T treatment (No CR on T treatment); and (4) No CR discontinued T treatment (No CR off T treatment). For descriptive purposes, the ‘CR on continuous T treatment group’ was further divided by PD status.

Person-time is the sum of follow-up time for all patients in the cohort [14]. Pearson chi-square test comparisons of categorical characteristics by CR and mean comparisons for continuous variables were used (F statistic). Two-sided test *p* values were used at 0.05 level of significance with Fishers exact testing for small cell numbers. Kaplan–Meier estimation was used to calculate 5-year DSS (log rank tests).

Covariates evaluated for association with complete response (CR) were used to build both logistic regression and Cox proportional hazard models with forced entry and forward stepwise methods. The multivariable Cox proportional hazards model was used to estimate adjusted hazard ratios (HzR) and corresponding 95% confidence intervals (CI) using DSS as the outcome. We evaluated the proportional hazards assumption by plotting ln{− ln(survival)} curves for the ordinal covariate of diagnosis year versus ln (at risk time) and on the basis of Schoenfeld residuals after fitting individual Cox models. We found no evidence suggesting substantial violation of the proportionality assumption graphically or in tests for interaction with the logarithm of survival time [15].

IBM SPSS v28 was used for bivariate, regression and survival statistical analysis [16]. In regression models, the forward stepwise models used 0.05 significance for inclusion in the model at each step. Individual time intervals for trastuzumab treatment and outcomes were used to create a swimmer plot using R [17].

## Results

Thirty seven of the 69 patients in our HER2-positive de novo MBC cohort treated with trastuzumab as initial therapy had a complete response (54%). Mean age of cases in our cohort was 52 years, range 29 to 87 years with no difference in CR by age less than 65 years or 65 years or greater. The cohort was 74% white with no difference in CR by white/non-white. Hormone receptor status and hormone therapy did not differ by CR status. (Table 1).

**Table 1:** Descriptive characteristics HER2 + dnMBC by CR/No CR, 1999–2018 (*n* = 69)

	CR	No CR	
	(*n* = 37)	(*n* = 32)	*p* value
	*N* (%)	*N* (%)	
Age			
< 65	30 (81%)	25 (78%)	0.774^a^
65 +	7 (19%)	7 (22%)	
Mean age in years (range, F statistic)	50 (29–77)	55 (33–87)	0.201
Race			
White	29 (78%)	22 (69%)	0.418^a^
Non-White	8 (22%)	10 (31%)	
HR/HER2 status at initial diagnosis			
HR + /HER2 +	21 (57%)	15 (47%)	0.474
HR-/HER2 +	16 (43%)	17 (53%)	
Number of distant metastatic sites			
1–2	35 (95%)	24 (75%)	0.037^a^
3–5	2 (5%)	8 (25%)	
Initial Treatment			
Pre-operative chemotherapy = yes	22 (60%)	4 (13%)	< 0.001^a^
Surgery = yes	25 (68%)	7 (22%)	< 0.001^a^
Radiation therapy = yes	21 (57%)	13 (41%)	0.181
Chemotherapy = yes	35 (95%)	25 (78%)	0.071
Hormone therapy = yes	18 (49%)	14 (44%)	0.684
Switched to TDM1	2 (5%)	3 (9%)	0.657^a^
Mean years on trastuzumab (range, F statistic)	4.8 (.8–14.1)	3.6 (.3–15.0)	0.161
Mean survival years (range, F statistic)	7.1 (2.4–14.2)	4.7 (1.0–15.0)	0.003
Follow-up status			
Alive NED^b^	22 (60%)	0	< 0.001
Alive with this cancer	3 (8%)	16 (50%)	
Expired NED^b^	2 (5%)	0	
Expired with this cancer	10 (27%)	16 (50%)	

^a^Fishers exact test (2-sided)

^b^NED = no evidence of disease

Oligometastatic disease cases were more likely to have CR (95% vs 75%, *p* = 0.037). Pre-operative chemotherapy and surgery were also associated with CR. All pre-operative chemotherapy patients had breast surgery with the removal of any residual primary tumor. Six patients had surgery but did not have pre-operative chemotherapy for a total of 32 breast surgery patients, 46% of total. 92% of pre-operative chemotherapy patients were oligometastatic (24/26) at diagnosis.

In chi-square comparisons, CR did not differ by distant site(s) of metastases [bone (*n* = 15), liver (*n* = 16), lung (*n* = 7), distant lymph nodes (*n* = 9), brain (*n* = 1) or combination liver/lung/bone (*n* = 14), bone/lung/lymph nodes (*n* = 7)]. Distant metastases of liver-only patients had a better CR rate at 69% but it was not statistically significant. Sixty patients received either pre-operative or palliative chemotherapy. Of these, fifty-four received a taxane-based regimen with no difference in CR between those that did or did not receive a taxane. Thirty-nine patients received trastuzumab and 30 received trastuzumab and pertuzumab beginning in 2012 with no difference in CR between the two regimens.

Average patient follow-up time was 6 years, range 1–15 years, and 412 years total person-time for the cohort. Average time to CR was 9 months (range 1–27 months). Of these cases, one was CR NED after surgery and 1 month of trastuzumab treatment. Eight patients discontinued trastuzumab treatment after CR with three of the eight having subsequent PD and death from disease. Average time after CR to discontinuation of trastuzumab treatment for the five CR/off T treatment patient without PD was 24 months (range 6–86 months) and 0, 2 and 25 months for the three CR/off T treatment PD after CR patients. Average survival time for the five CR patients without PD was 10.6 years (range 7.6–14.2 years), all alive NED at last follow-up. Average survival was 6.0 years (range 2.7–7.8 years) for the three CR/off T treatment/PD patients, all died with disease. Of the 29 patients with CR who stayed on trastuzumab treatment, 45% had PD (13/29) with average DOR post CR 30 months (range 7–92 months) and seven died of disease. Two of the thirteen CR/on T treatment/PD patients had a subsequent 2nd CR after TDM1 treatment and were alive NED at last follow-up***.***

**Fig. 2:**
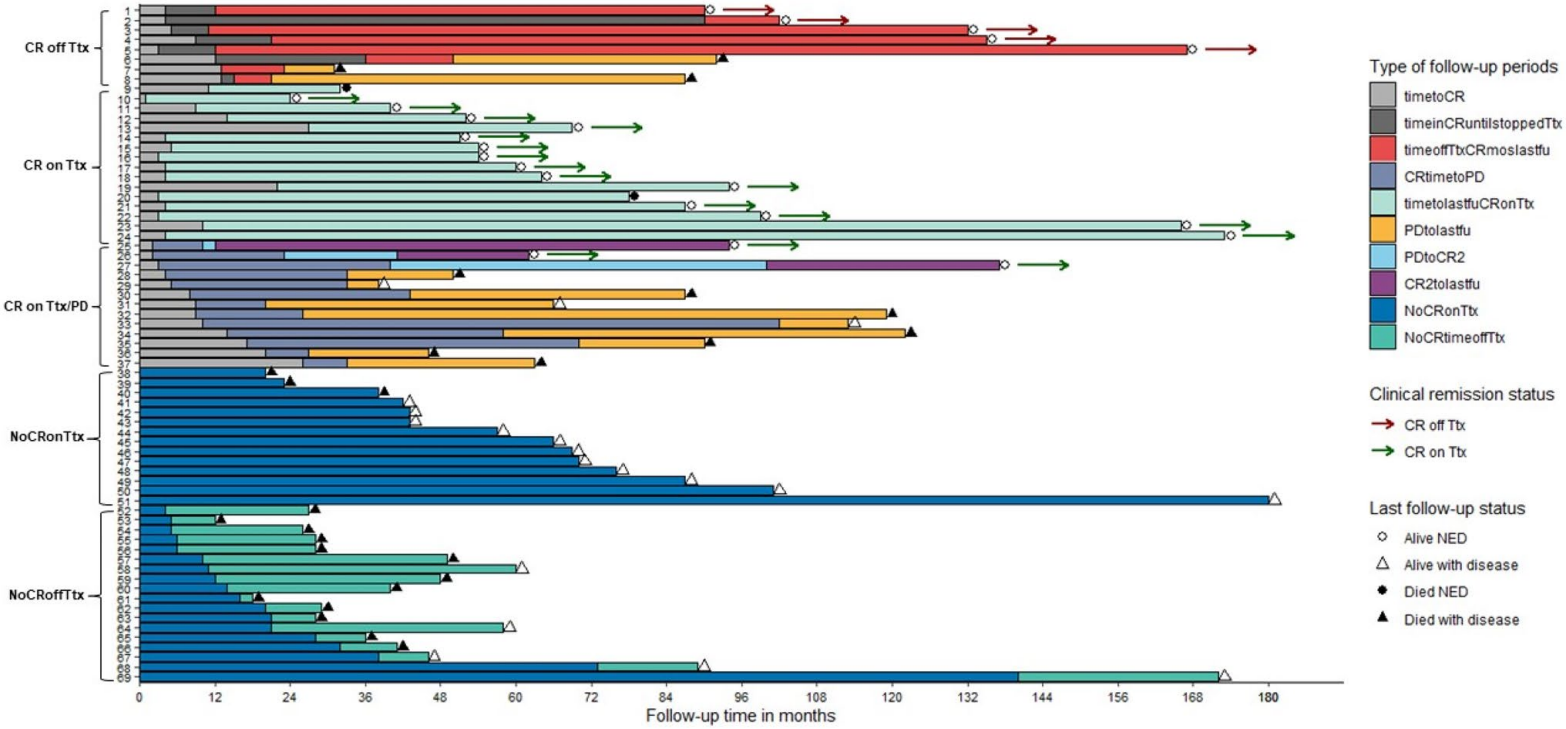
Swimmer plot of trastuzumab duration and patient outcomes

A portion of cases in each of the four treatment/outcome categories had survival of 5 or more years and 10 or more years: [(1) CR off T treatment: mean = 8.9 years (2.7–14.2 years), 88% 5 + years, 38% 10 + years; (2) CR on T treatment: mean 6.6 years (2.4–14.2), 66% 5 + years, 14% 10 + years; (3) No CR on T treatment: 5.5 years (1.7–15.0), 50% 5 + years, 7% 10 + years; (4) No CR off T treatment: mean 4.0 years (1.0–14.4), 18% 5 + years, 6% 10 + years]. In total, 28 patients (41%) survived 5 or more years post diagnosis (*p* = 0.004). (Fig. 2: swimmer plot).

In the forward stepwise logistic regression model for CR outcome with a 0.05 level of significance for inclusion in the model, only pre-operative chemotherapy was significant [OR = 10.27 (95% CI 2.98, 35.34) *p* < 0.001]. Variables in the model were age < 65/65 + , race white/non-white, number of distant sites 1–2/3 + , hormone receptor positive yes/no, pre-operative chemotherapy yes/no, adjuvant chemotherapy yes/no, radiation therapy yes/no, surgery yes/no, and hormone therapy yes/no.

**Fig. 3:**
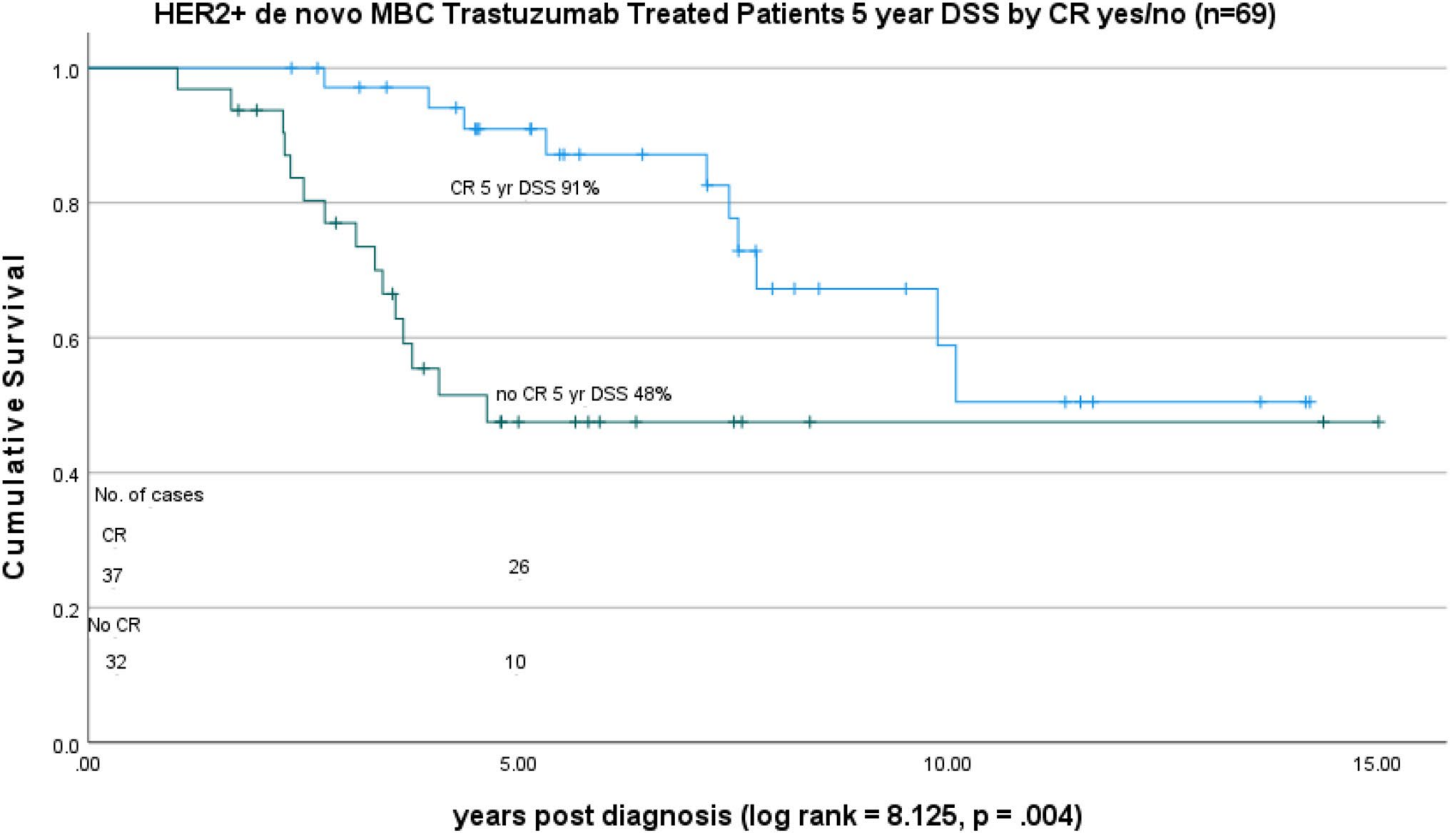
HER2 positive de novo MBC trastuzumab-treated patients 5-year DSS by CR status (yes/no) (n=69)

**Fig. 4:**
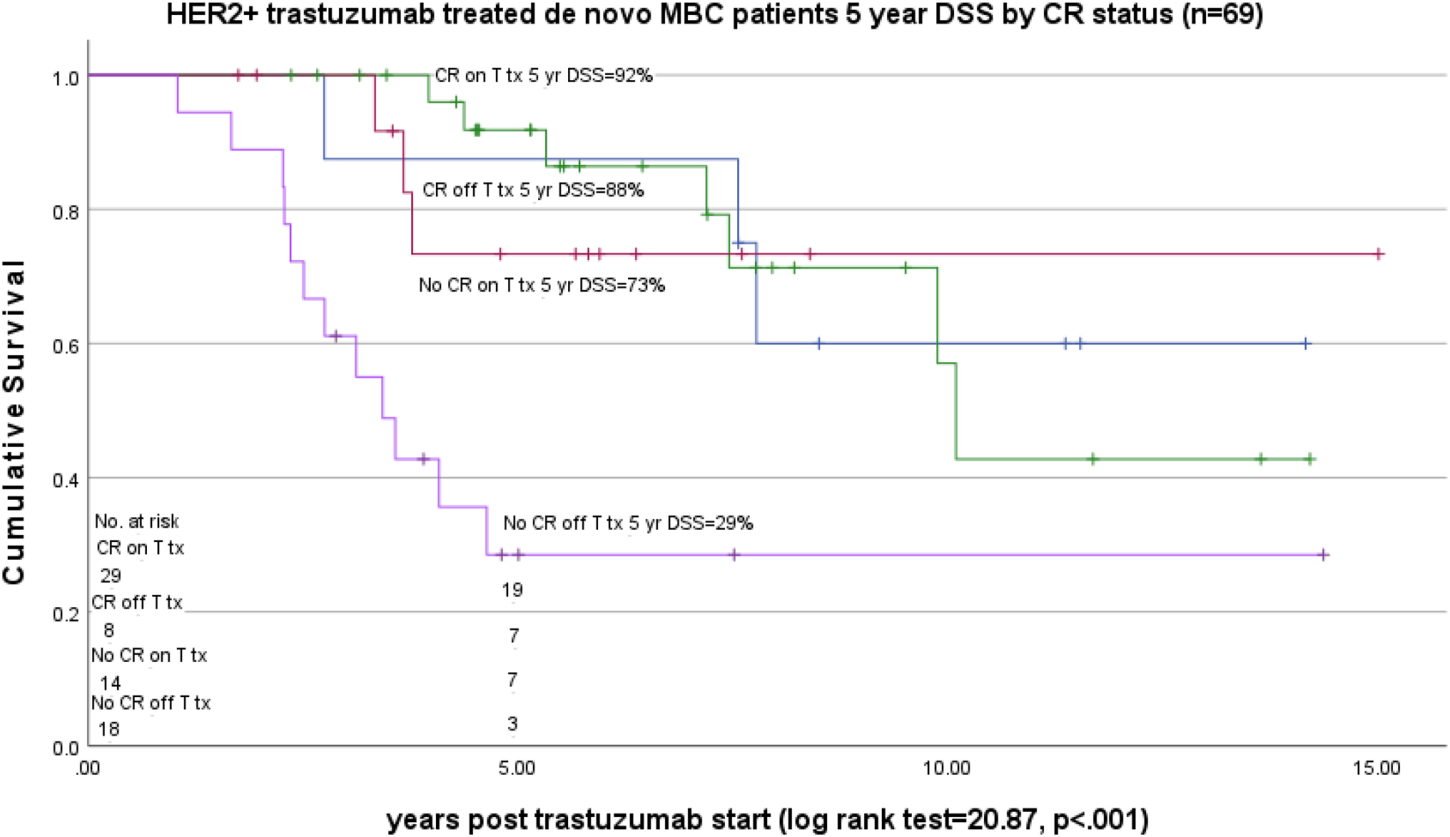
HER2 positive de novo MBC trastuzumab-treated patients 5-year DSS by 4 category CR/T treatment status (n=69)

In a Kaplan–Meier plot of 5-year disease-specific survival (DSS) by CR, DSS was 91% for patients with a CR and 48% for patients without a CR. (Fig. 3) In a Kaplan–Meier plot of 5-year disease-specific survival (DSS) by CR/trastuzumab treatment status, 5-year DSS was 92% CR on T (*N* = 29), 88% CR off T (*n* = 8), 73% No CR on T (*n* = 14), and 29% No CR off T (*n* = 18) (overall log rank comparison = 20.87, *p* < 0.001). The treatment/outcome groups, CR on T treatment, CR off T treatment, and No CR on T treatment had statistically better survival than No CR off T treatment but were not significantly different from each other. In the pairwise comparisons, only the No CR/off T treatment group was significantly different from the other three categories of CR/T treatment. (Fig. 4).

In the Cox proportional hazards enter model for outcome DSS, age less than 65/greater than 65, race white/non-white, hormone receptor status positive/negative, number of distant sites 1–2/3 + , pre-operative chemotherapy yes/no, surgery yes/no, chemotherapy yes/no, radiation therapy yes/no, complete response yes/no, and duration of trastuzumab treatment < 2/ ≥ 2 years were used. Younger age, CR, and T treatment ≥ 2 years were significant. (Table 2) In the Cox proportional hazards forward stepwise model with the same variables, 0.05 entry *p* value, CR = yes, continuous trastuzumab treatment ≥ 2 years, and age < 65 were significantly associated with better DSS [CR = yes: HzR = 0.31, (95% CI 0.14, 0.73), *p* = 0.007; T treatment ≥ 2 years: HzR = 0.24, (95% CI 0.10, 0.62), *p* = 0.003; age < 65: HzR = 0.29, (95% CI 0.11, 0.81), *p* = 0.018]. (Table 2).

**Table 2:** Cox proportional hazards model HER2 + dnMBC patients: DSS (*n* = 69)

	HzR (95% CI)	*p* value
Method: enter		
Age		
65 + years	Reference	
< 65 years	0.31 (0.11, 0.85)	0.023
Race		
White	Reference	
Non-white	1.37 (0.47, 3.95)	0.563
Number of distant sites		
3 +	Reference	
1–2	0.59 (0.17, 2.10)	0.414
Hormone receptor-positive		
Positive	Reference	
Negative	2.25 (0.83, 6.09)	0.112
Pre-operative chemotherapy		
No	Reference	
Yes	0.36 (0.07, 1.89)	0.230
Adjuvant chemotherapy		
No	Reference	
Yes	1.08 (0.25, 4.70)	0.914
Surgery		
No	Reference	
Yes	4.14 (0.97, 17.64)	0.055
Radiation therapy		
No	Reference	
Yes	0.82 (0.28, 2.41)	0.720
Complete response		
No	Reference	
Yes	0.22 (0.07, 0.67)	0.008
Trastuzumab treatment		
< 2 years	Reference	
> 2 years	0.30 (0.12, 0.77)	0.013
Method: forward stepwise		
Age < 65 years	0.29 (0.11, 0.81)	0.018
Complete response = yes	0.24 (0.10, 0.62)	0.003
Trastuzumab treatment ≥ 2 years	0.31 (0.14, 0.73)	0.007

## Discussion

More than half of patients had CR with time to CR averaging 9 months and all achieving CR by 27 months. CR patients were more likely oligometastatic (1–2 metastatic sites), had pre-operative chemotherapy and/or surgery. Few patients went off trastuzumab treatment after CR with most staying on maintenance trastuzumab. Seventy percent of complete responses lasted more than a year and CR patients had better survival. There were patients with extraordinary survival beyond 5 and 10 years in each treatment/outcome group, including patients who never attained CR. In adjusted Cox proportional hazards modeling, continued trastuzumab treatment past 2 years, younger age, and CR were independently associated with better disease-specific survival.

For the 69 patients, of the 37 patients who achieved CR, eight went off trastuzumab treatment with three of the eight relapsing and dying. Given this risk of relapse, it is unclear if, and when, trastuzumab maintenance therapy can safely be discontinued. Ninety-two percent of pre-operative chemotherapy-treated patients with subsequent surgery were oligometastatic, a situation with lower disease burden contributing to better outcomes. It appears trastuzumab has considerable potential benefit in favorable metastatic situations when CR is achieved or in the absence of progression and continued trastuzumab treatment.

### Comparison of results to other published studies

Few studies of exclusively de novo MBC exist. Wong et al. found 13% of 483 HER2-positive de novo MBC patients achieved NED status compared to 54% in our smaller cohort [3]. In their study, NED status was associated with number of metastatic sites 1 vs > 1 and surgery. Other studies combined both de novo and recurrent MBC patients. Steenbruggen et al. found a 10% rate of CR in their cohort (*n* = 717) with CR status associated with survival [7]. Thirty patients who achieved CR discontinued trastuzumab therapy after an average of 6 months with twenty experiencing ongoing remission at median follow-up of 78 months. Gelmon et al. observed a 39% overall response rate to trastuzumab alone or with a taxane as first regimen in a retrospective case review of HER2 - positive MBC patients [18]. Battisti et al. studied HER2-positive MBC patients (*n* = 208) who received at least 1 year of first-line anti-HER2 therapy, seventy-nine of which had de novo MBC [19]. The CR rate was 21% overall. Seven of the CR patients with prolonged CR electively discontinued therapy at an average of 62 months with all seven alive in remission at an average of 112 months. Outcomes for the 37 CR patients remaining on therapy were not reported. Schmid et al. found durable clinical benefit from trastuzumab monotherapy to be predictive of longer overall survival in HER2-positive advanced disease [20]. In a study of patients with at least 2 years first-line trastuzumab treatment, of those with a clinical complete response, four of twenty-five with T treatment interruption had progressive disease [21]. The 54% rate of CR observed in our cohort study was higher than other cohort studies of trastuzumab-treated HER2-positive MBC but our cohort was exclusively first primary treatment naive HER2-positive de novo MBC.

Individual case reports and real-world studies of prolonged HER2-positive MBC remission with trastuzumab treatment are available but whether a patient can go off T treatment and retain remission status has scant evidence [22–25]. Gullo et al. reviewed durable complete response for 5- vs. 2-year trastuzumab-treated HER2-positive MBC patients at two institutions with no statistically significant difference between the two groups for durable response [26]. 8/53 (15%) patients who received 5 years had CR, six of which were durable (11%). 5/31 (16%) patients who received 2 years of therapy had CR, of which two were durable. With long-term follow-up, a portion of the HER2 -positive MBC patients had prolonged complete remissions without statistical significance between the two treatment times. Tarantino et al. suggest a HER2-positive MBC tailored cure is possible with de novo status and oligometastatic disease related to long-term response which may be supported by our observations [27]. High HER2 amplification levels and the absence of detrimental genomic aberrations, PIK3CA and PTEN, were also related to long-term response in that study.

For HER2-positive invasive breast cancer patients with progressive disease, the sequence of subsequent treatments has not been determined. Prior to the development of the antibody drug conjugates (ADCs) T-DM1 and trastuzumab deruxtecan (T-Dxd), there was considerable debate regarding whether or not to continue trastuzumab while changing hormone and/or chemotherapy [23, 25, 28, 29]. Current ADC treatment trials in both adjuvant (KATHERINE trial) and metastatic settings (DESTINY03 trial) show promise for alternative HER2-positive breast cancer treatment beyond trastuzumab and standard chemotherapy [30, 31]. In our study, of the 25% of patients who did not have a CR with trastuzumab treatment and discontinued trastuzumab, three patients had T-DM1 treatment without success. The utility of trastuzumab-containing regimens after progression on ADCs requires exploration as well.

None of our patients had reported cardiotoxicity, though this may be due to small sample size. The risk of cardiotoxicity from trastuzumab is primarily confined to the first 2 years of treatment [32]. To our knowledge, no new safety signals have been reported with prolonged therapy. Therefore, duration of trastuzumab therapy does not require a time limit for fear of increased cardiotoxicity [33].

The 1-year recommended length of adjuvant trastuzumab for patients with localized disease is well established [5]. Efficacy of both shorter and longer adjuvant trastuzumab has not been confirmed [34–36]. To date, prospective studies of HER2-positive de novo MBC have not assessed length of effective treatment time for clinical response, treatment time after CR for durable response or continuation of treatment with or without progression. Current characterization of effective MBC trastuzumab treatment time relies on real-world studies and possibly follow-up analysis of clinical trials. Randomized clinical trials to address trastuzumab maintenance duration and treatment switching strategies with and without trastuzumab continuation in the metastatic setting would be welcome although possibly difficult to conduct with the question of whether to remove a potentially life-saving treatment. We anticipate the results of the proposed STOP HER2 trial of trastuzumab discontinuation in HER2-positive MBC long-term responders who are progression-free after 3 or more years on first-line anti-HER2 therapy with close follow-up and extensive biomarker assessment [37].

### Strengths and limitations

The primary strength of our study is the detailed observation of HER2-positive de novo MBC patients over an extensive time period with treatment, response, progression, and vital status recorded with follow-up at regular intervals. Even with this level of detail and support, a retrospective cohort study does not have the same level of evidence as a randomized clinical trial. A small patient cohort such as ours even with extensive follow-up years precludes some detailed treatment analysis. However, the number of patients was adequate for basic factor analysis as evidenced by the tight 95% confidence intervals in the statistical models.

In our retrospective cohort, we found CR occurred within 2 years of trastuzumab treatment initiation adjusted for other treatments which could relate solely to continued trastuzumab or to companion therapy including surgery or radiation. In our adjusted Cox proportional hazards model, continued trastuzumab treatment had a statistically significant independent favorable effect on survival. Progressive disease occurred in both those staying on treatment and those going off but larger numbers are needed to assess statistical significance.

## Summary and conclusions

Persistent questions for HER2-positive de novo MBC patient care are how long trastuzumab should be continued in CR patients and if it should be continued after disease progression while changing companion therapy given other treatment options. Our finding of disease progression in both CR patients who discontinued and continued trastuzumab treatment after CR indicates other unmeasured factors involved in the disease process. The durable treatment success for some patients who went off trastuzumab therapy with no other continued therapy after CR holds promise. Better disease-specific survival related to younger age, complete response, and longer trastuzumab treatment present factors to consider when making treatment duration decisions. New studies of minimal residual disease presence through analysis of circulating tumor DNA (ctDNA) and DNA methylation in plasma may help guide these decisions [38, 39]. Going forward the development of multiomic analysis of plasma as well as new, effective HER2-specific treatment options may also influence treatment decisions [30, 31, 40, 41].

Our finding of HER2-positive MBC survival after both 5- and 10-years post diagnosis time indicates a stable disease state may be achievable, for patients either alive with disease or clinically NED, with appropriate therapy. It appears treatment with curative intent could be considered for HER2-positive de novo metastatic breast cancer patients.

## Data Availability

The dataset analyzed during the current study is not publicly available due to HIPAA Security Rules regarding patient data at the institution where the registry was created and is kept and is not available from the corresponding author on reasonable request.
